# Editorial: Mycorrhizosphere Communication: Mycorrhizal Fungi and Endophytic Fungus-Plant Interactions

**DOI:** 10.3389/fmicb.2018.03015

**Published:** 2018-12-05

**Authors:** Erika Kothe, Katarzyna Turnau

**Affiliations:** ^1^Institute of Microbiology, Friedrich Schiller University, Jena, Germany; ^2^Institute of Environmental Sciences, Jagiellonian University in Krakow, Kraków, Poland

**Keywords:** mycorrhiza, endophyte, fungus-plant interactions, mycorrhizophere, communication

## Plant Mycobiomes

Plants do not exist as single entities but should rather be considered to form a complex community with microbes and other organisms where plant tissues form diverse niches for microbes. One major relationship concerns plant-fungal interactions that range from pathogenicity to mutually beneficial symbioses. A balanced state (homeostasis) of these interactions is essential for maintaining the plant as well as an overall healthy state of the environment. Mycorrhizal associations are well-studied examples of root-fungal mutually beneficial symbiosis (Ferlian et al., 2018; Gehring and Johnson, 2018). The reasons for establishing a mutual symbiosis are only just beginning to be understood at the molecular level (Mello and Balestrini). Communication between endo- and ecto-mycorrhiza and their respective host plants (Raudaskoski and Kothe, 2015; Luginbuehl and Oldroyd, 2017; Garcia et al., 2018, and citations therein) and the effects on phytohormone levels and localized delivery (see Boivin et al., 2016; MacLean et al., 2017) have been the focus of several recent reports. But even so, a full understanding of these relationships will only be gained by investigating the effects of different strains of the same fungal species (Sharma et al.).

For roots, as well as above-ground plant tissues, endophytic fungi can be considered as examples of specific co-evolution, provided the term “endophytic” is used in its *sensu strictu* (for a detailed comment, see Kothe and Dudeja, 2011). To prove endophytic behavior, Koch's postulates need to be observed, and tissue specificity for re-infection may be used as a method to discriminate real endophytes from mere co-occurrence (Wȩżowicz et al., 2017; Domka et al., 2018; Ważny et al., 2018). The traits of endophytes that do not lead to symptoms in a healthy plant clearly delineate them from phytopathogenic fungi, however, caution is necessary because their effect on the symbiosis can vary with the species/variety of the partner and environmental conditions. For endophytic fungi, knowledge is much more limited as compared to mycorrhiza, although a role for strigolactone signaling is presented by Rozpądek et al.

Temporal shifts in plant-associated fungal populations are known to occur. An example from mycorrhizal symbiosis is for young trees with endomycorrhizal symbionts that are later replaced by specific ectomycorrhizal associations (Knoblochová et al., 2017; Bachelot et al., 2018). Within 4 weeks, vesicles and hyphae are visible in the roots of *Picea abies* and *Pinus sylvestis* leading to increased main-root development and up to a 300% increase in secondary roots. These effects alone will increase the potential at a later stage for formation of ectomycorrhiza, which is the only form of mycorrhiza seen in mature pine and spruce. After the ectomycorrhiza is established, a succession of fungal partners appears. First, fast-growing, broad host-range, reproduction-strategy fungi are attracted; later, slower growing, but more specific, ectomycorrhizal fungi, acting by capacity strategy, are recruited. For spruce, that would lead to replacement of, e.g., *Cenococcum geophilum* (see de Freitas Periera et al.) or *Pisolithus tinctorius* by host-specific fungi like *T. vaccinum*.

## Multi-Omics in Fungal-Plant Molecular Communication

The mycobionts and their hosts will constantly communicate to establish and maintain the symbiosis. Signals are perceived and result in changes in gene expression. With excreted proteins or metabolites, the partner is stimulated. A multi-level interaction thus will be visible with changed transcriptome, proteome, and metabolome patterns. These can be visualized with techniques such as transcriptomics (Fiorilli et al., 2016; Nagabhyru et al., 2018), proteomics (Sebastiana et al., 2017; Shrivastava et al., 2018), metabolomics (Hill et al., 2018; Maciá-Vicente et al., 2018), or combinations thereof (e.g., Larsen et al., 2016). Since secreted proteins may be important for the signal exchange, secretomics can be expected to identify effector proteins exchanged between symbiont and host (Doré et al., 2015; Wagner et al., 2015). Volatiles exchanged address a subgroup of metabolites for signal exchange (Ditengou et al., 2015; Pistelli et al., 2017). And a combination of translatome and proteome data (Sharma et al.), as well as multi-omics have proven to substantially improve the quality of prediction for the symbiotic molecular network (Vijayakumar et al., 2016).

Interactions between symbiotic fungus and plant in production of secondary metabolites (Ludwig-Müller, 2015; González-Menéndez et al., 2016) shows the intricate relationship between the symbiotic partners. In addition to the dual interaction, the communication is expanded with additional fungi or bacteria which co-occur in the environment.

## Multi-Partner Interactions

Usually, plant and fungus are not alone in the partnership, but additional interactions with bacteria or other fungi will influence the outcome of these associations in nature. This complex relationship is reflected in the concept of the mycorrhizosphere, where plant roots and hyphae of the mycorrhizal partner encounter other soil microorganisms. These additional interactions in the vicinity of the root need to be considered in studies of cross-talk (Vannini et al., 2016; Wagner et al., 2016).

Phytohormones produced in the mycorrhizosphere may alter the physiology of the symbiotic partners and aid formation of new mycorrhiza (Wagner et al., 2015). For example, the ectomycorrhizal *Tricholoma vaccinum* produces the auxin indole-3-acetic acid (IAA), which stimulates mycorrhization (Krause et al., 2015). In addition, the exogenous presence of the phytohormone promotes branching, which leads to an increased Hartig-net formation during symbiosis (Krause et al., 2015). Moreover, ectomycorrhization can be reversed by associated fungi that produce IAA-inhibiting compounds (Hause and Saarschmidt, 2009). With soil zygomycetes, a tripartite interaction occurs in which the zygomycete-derived metabolite, D-orenone, induces a transporter that allows for increased excretion of IAA by the mycorrhizal fungus, *T. vaccinum* (Wagner et al., 2016). Soil bacteria are also capable of auxin biosynthesis, mostly upon tryptophan induction *via* root exudates. Since IAA increases branching of ectomycorrhizal fungi, some have been termed mycorrhiza-helper bacteria (Frey-Klett et al., 2016). It is now clear that multi-partner communication systems have evolved and are present in any habitat.

Like symbionts, phytopathogens also act *via* phytohormes, e.g., by inducing systemic-acquired resistance in host plants (Scholz et al.). Similar mechanisms of cross-talk can be inferred from comparison of pathosystems to endophytic or mycorrhizal symbiosis.

## Molecular Responses to Environmental Stresses

In addition to biotic interactions shaping the plant mycobiome, abiotic factors certainly influence the mutual, commensal or pathogenic interaction. Environmental conditions may influence plant mycobiomes, and these effects are more likely observed under detrimental conditions, such as nutrient limitation, drought, salinity, or other stresses, for example metal toxicity (Kumar and Verma, 2018; Shi et al., 2018). The molecular background of stress recognition and signal transduction within endomycorrhiza is reported by Sun et al. and an example of endophytes altering phosphate mobility in the mycorrhizosphere is given by Baum et al. The molecular background of stress recognition and signal transduction with an endomycorrhizal association is reported by Sun et al. as is an example of endophytes changing phosphate mobilities in the mycorrhizosphere (Baum et al.).

Environmental stresses, like metal contamination in the ground, to name just one example, can be buffered by mycorrhizal and endophytic associations. A molecular role has been shown for hydrophobins, small amphipatic proteins that decorate the cell wall of air-exposed mycelium. A study by Sammer et al. on the up-regulation of different hydrophobins during the life cycle of the mycorrhizal fungus exposed to metal contamination illustrates their protective effect. In that study, the biotic interaction is also characterized by volatiles and exudates from the host tree inducing mycorrhiza-associated hydrophobin genes (Sammer et al., 2016).

Transporter production, as well as intracellular storage of metals, illustrate specific adaptive responses in fungi. Many fungi are able to carry increased metal loads if grown on metal-contaminated substrate. A prominent example is the mushrooms that showed high cesium content when collected from the fall-out areas after the Chernobyl accident. A molecular explanation is now available for this observation (see Benes et al.). For metals that are not only toxic, but essential for growth at lower concentrations, e.g., zinc, require the presence of a specific uptake and storage system. Indeed, for zinc, such a system has been described with the ectomycorrhizal fungus *Suillus luteus* (Coninx et al.). As an additional twist, there are fungi that influence mutual symbioses of bacteria with plants. An example of this is shown by Thiem et al. where the influence of microbiome, including the mycobiome, on the actinorrhizal interaction between *Frankia* and alder (*Alnus glutinosa*) under salinity is demonstrated.

## Applications for Growing Demands on Sustainable Agriculture and Forestry

Anthropogenic impact, including industrial pollution and both conventional and organic agriculture, has already affected the soil microbiome, leading to decreases in soil quality and the nutritional value of crops. In doing so, it has created the necessity to use a range of chemicals, such as fertilizers and pesticides, to avoid the spread of unwanted pathogenic microbes. These substances not only affect plant-microbial foes but also the friendly microbes that help the plant to establish homeostasis and attain the nutritional quality of products that support the health of the consumers. The twenty first century brings us possibilities to develop new and innovative methods to rebuild soil tilth and to renovate the plant microbiota (see, e.g., Verzeaux et al., 2017; Campos et al., 2018). However, to be successful, we need an increased understanding by both food and wood producers on the molecular communication between fungi and the host plant, resulting in competitive advantages specifically under abiotic stress. The results may provide solutions for the problems aggravating sustainable agriculture and forestry, especially under the ever-changing environmental conditions (Shinde et al., 2018).

The re-introduction of protective microbes can be achieved through bioaugmentation strategies, which allow them to create optimal conditions for crop growth under harsh conditions (see Treu and Falandysz, 2017) and a reduction in the use of water, fertilizers, and pesticides. The most common plant inhabitants are endophytes that, when properly selected, can be a potent tool against pathogens and abiotic factors (see French, 2017). They also support mycorrhizae, which in turn contribute to plant growth and induce tolerance to salinity, pollution, drought, extreme temperatures, elevated CO_2_, etc. (see, e.g., Dhawi et al., 2017). The provision of well-selected microbes and the application of appropriate agricultural/forestry practices, both of which are feasible now, can decrease the current intensive use of fertilizers while maintaining an environment that is conducive to human health. Although nowadays the interaction of fungi and bacteria with plants is better understood, there is still a need for greater insight into the interplay between bacteria and fungi, fungi with other fungi, and their interactions with plants. Furthermore, building stronger bridges between bacteriologists and mycologists will help to benefit from their complementary skills, as exemplified by the work on the roles of strigolactones (see, De Cuyper and Goormachtig, 2017).

## Author Contributions

All authors listed have made a substantial, direct and intellectual contribution to the work, and approved it for publication.

### Conflict of Interest Statement

The authors declare that the research was conducted in the absence of any commercial or financial relationships that could be construed as a potential conflict of interest.
